# Editorial: Plant secondary metabolites and their effects on environmental adaptation based on functional genomics

**DOI:** 10.3389/fgene.2023.1211639

**Published:** 2023-05-16

**Authors:** Zishu Xu, Najeeb Ullah, Yi Duan, Zhuoni Hou, Ake Liu, Ling Xu

**Affiliations:** ^1^ Key Laboratory of Plant Secondary Metabolism and Regulation of Zhejiang Province, College of Life Sciences and Medicine, Zhejiang Sci-Tech University, Hangzhou, China; ^2^ Agricultural Research Station, Office of VP for Research and Graduate Studies, Qatar University, Doha, Qatar; ^3^ Department of Life Sciences, Changzhi University, Changzhi, China

**Keywords:** plant secondary metabolites (PSMs), stress tolerance, functional genomics, molecular adaptation, transcription factors

## Introduction

Secondary metabolites are organic compounds produced by plants that are not directly involved in growth, development, or reproduction, but play important roles in adaptation to the environment (Hu et al., 2018). These compounds have diverse chemical structures and biological functions, such as defense against herbivores and pathogens, the attraction of pollinators, and regulation of growth and development (Moore et al., 2014; Yuan et al., 2022). Understanding the genetic basis of secondary metabolite biosynthesis and their ecological functions is crucial for developing sustainable agriculture and protecting natural ecosystems (AbdAlla et al., 2023). For example, how do plants regulate secondary metabolite synthesis and how do plant secondary metabolites (PSM) influence environmental adaptation? In the past, it was generally thought that PSMs were small biological molecules and non-essential for organism survival (Speed et al., 2015; Li et al., 2020; Shi and Du, 2023). However, it is extensively accepted that PSMs play essential roles in environmental adaptation by providing protection against environmental stressors and supporting the growth of symbiotic organisms (Li et al., 2021; Xu et al., 2022; Makhumbila et al., 2023).

Recent advances in functional genomics have enabled researchers to identify and characterize the genes and enzymes involved in secondary metabolite biosynthesis in various plant species (Meng et al., 2023). Modern analytical tools such as transcriptomics, proteomics, and metabolomics have been used to identify the genes that are upregulated in response to environmental stresses, however, a mechanistic understanding of the stress response is still emerging (Khodavirdipour et al., 2022; Wei et al., 2022; Yuan et al., 2022). The number of novel variations is expected to increase as biotechnology and genome/transcriptome sequencing technologies progress, and how to identify and use functional genes in enhancing tolerance is of paramount importance both now and in the future (Liu et al., 2022; Niu et al., 2022; Meng et al., 2023). In this Frontiers Research Topic, key questions related to PSMs are addressed by revealing essential genes and mechanisms. The effects of PSMs on environmental adaptation are also discussed by 29 authors.

## Effects on environmental adaptation of plant secondary metabolites

Drought, heat, and cold are abiotic environmental factors that constantly stress plants, which can seriously affect the quality and yields of crops. Plants mitigate damage from stress in various ways, including modulating the synthesis of PSMs and expression of genes. Zhao et al. found that poplars (*Populus* spp) enhance the tolerance to heat by increasing lignin contents. Moreover, they found that poplar plants respond to heat stress by altering key regulators and metabolic intermediates. For example, through an enhanced biosynthesis of caffeate and coniferaldehyde, poplar plants increase lignin accumulation in stems to withstand high temperature stress. Similarly, Dong et al. described a series functions of B-box transcription factor genes in sweet potato (*Ipomoea batatas* (L.) Lam.), especially *IbBBX28*. They found a negative association between IbBBX28 and drought resistance in plants. This provides the foundation for future studies about the role of B-box transcription factors in the resistance of sweet potato under stress.

## Functional genes of plant secondary metabolites

The exploration of functional genes is crucial to better understanding how plants regulate the biosynthesis of their secondary metabolites, in order to analyze the molecular mechanisms underlying this process. Concerning the PSMs of essential oil, Hou et al. investigated the molecular mechanism that modulates the production of monoterpenes and sesquiterpenes in *Cinnamomum burmannii’*s secondary metabolites. They found the origins of essential oil in *C. burmannii*, and further explored the differentially expressed genes and lncRNAs that were associated with the synthesis of monoterpenoids and sesquiterpenoids during the leaf development of *C. burmannii*. They also offered a novel insight into the mechanisms of PSMs synthesized in *C. burmannii*. Furthermore, Huang et al. reported that several WRKY transcription factors are involved in protoberberine alkaloids biosynthesis in *Coptis chinensis* Franch, were also observed subcellular. Further investigation of berberine biosynthesis regulation may be based on these findings. Zhao et al. also analyzed the candidate transcription factors associated with lignin biosynthesis under heat stress.

Throughout the history of plant research, each generation has explored a series of functional genes associated with PSMs. These explorations of functional genes promote the past, present, and future cash crop breeding. These four studies presented provide insight into how the functional genes regulate PSM biosynthesis and in turn the secondary metabolites enhance plant environmental adaptability. Future studies will need to revisit and solve fundamental mechanisms of PSM biosynthesis and environmental adaptation in order to effectively breed cash crops with better tolerance to multiple stresses.
